# Supplementary material for: Engineering precursor pools for increasing production of odd-chain fatty acids in Yarrowia lipolytica
Source: Metab Eng Commun. 2020 Dec 19;12:e00158. doi: 10.1016/j.mec.2020.e00158 (PMC7773535; doi:10.1016/j.mec.2020.e00158)

**Supplementary material**

**Engineering precursor pools for increasing production of odd-chain fatty acids in *Yarrowia lipolytica***

**Young-Kyoung Park^1*^, Florence Bordes^2^, Fabien Letisse^2,3^, Jean-Marc Nicaud^1^**

^1^ Université Paris-Saclay, INRAE, AgroParisTech, Micalis Institute, Jouy-en-Josas, France

^2^ Toulouse Biotechnology Institute, Université de Toulouse, CNRS, INRAE, INSA, Toulouse, France

^3^ Université Toulouse III - Paul Sabatier, Toulouse, France

**Supplementary Table 1.** The primers used in this study.

| Name | Sequence (5’ – 3’) |
| --- | --- |
| pTEF-internal-Fw | TCTGGAATCTACGCTTGTTCA |
| ACS2-Fw | ATGTCTGAAGACCACCCAGC |
| ACS2-intern-Rev | GCTCGTGGGTGTTCCACA |
| CpPCT-intern-Rev | GCAACAGAAGCCACGTACTCA |
| EnPCT-Fw | ATGACCCACCCCCAGCAG |
| EnPCT-internal-Rev | GACCGGCAGATCCAGGTCGG |
| EcPCT-Fw | ATGAAACCTGTCAAACCGCC |
| EcPCT-internal-Rev | GATTCCTTGCGAAGTGATTCCG |
| RePCT-Fw | ATGAAGGTGATTACCGCCAGAG |
| RePCT-internal-Rev | CCAATGGGGCCTGCCTC |
| EcPrpE-Fw | ATGTCTTTCTCCGAGTTCTACCAG |
| EcPrpE-Rev | CTACTCCTCCATAGCCTGTCG |
| SePrpE-Fw | ATGTCTTTCTCCGAGTTCTACCAG |
| SePrpE-Rev | CTACTCCTCAATGGCCTGTCG |
| ReBktB-Fw | ATGACCCGAGAGGTGGTGGTGGTC |
| ReBktB-internal-Rev | CCTTGAAGTAGCCGGCCTTGATGG |
| ReBktB-Rev | GATTCGCTCGAAGATGGCAGCGATG |

**Supplementary Table 2.** The sequences of the genes used in this study.

| Name | Sequence (5’ – 3’) |
| --- | --- |
| *Repct* | ATGAAGGTGATTACCGCCAGAGAAGCAGCGGCTCTTGTGCAGGACGGTTGGACTGTTGCATCGGCTGGATTCGTTGGCGCAGGCCATGCTGAGGCAGTCACCGAAGCCCTTGAGCAGCGATTTCTGCAATCGGGTCTGCCACGAGATCTGACCCTCGTCTACTCTGCTGGACAGGGCGATCGTGGTGCCCGAGGTGTGAACCACTTCGGCAATGCCGGCATGACCGCCAGCATCGTCGGCGGCCATTGGAGATCCGCAACCAGACTCGCCACCCTGGCCATGGCTGAGCAGTGTGAGGGCTACAACCTGCCTCAAGGCGTCCTTACGCACCTGTACCGAGCCATTGCTGGCGGTAAACCTGGTGTCATGACCAAGATCGGCCTCCATACGTTCGTCGACCCACGAACCGCCCAAGATGCCCGATACCATGGCGGCGCGGTTAACGAGCGAGCACGGCAGGCCATTGCCGAGGGAAAGGCTTGCTGGGTTGACGCCGTGGACTTTCGAGGCGATGAGTACCTGTTCTACCCCTCGTTTCCCATCCACTGTGCGCTCATTCGGTGCACTGCCGCTGACGCCCGAGGAAACCTCTCCACTCACAGAGAGGCCTTTCACCACGAACTTTTGGCAATGGCCCAAGCTGCTCACAACTCCGGAGGCATCGTCATCGCGCAGGTGGAGTCCCTCGTGGACCACCACGAGATTCTGCAGGCCATCCACGTTCCAGGCATTCTGGTGGACTACGTCGTGGTTTGCGACAACCCCGCTAATCACCAGATGACCTTCGCCGAGTCCTACAACCCTGCGTACGTCACGCCTTGGCAGGGAGAAGCTGCCGTGGCCGAAGCCGAGGCCGCTCCCGTCGCTGCTGGACCCCTTGACGCGCGGACCATCGTGCAGCGTCGAGCCGTTATGGAGCTGGCCCGACGAGCCCCGCGAGTTGTGAACCTCGGTGTCGGAATGCCTGCTGCCGTTGGTATGCTCGCCCATCAGGCTGGACTCGACGGCTTCACCCTGACTGTGGAGGCAGGCCCCATTGGTGGTACTCCCGCTGACGGACTGTCCTTTGGTGCCTCTGCTTATCCGGAGGCTGTCGTCGACCAGCCTGCCCAGTTCGACTTCTACGAAGGCGGTGGCATTGACCTTGCCATCCTCGGCTTGGCTGAGCTCGATGGTCACGGCAACGTCAACGTGTCCAAGTTCGGTGAGGGAGAAGGAGCCTCCATTGCTGGTGTTGGCGGTTTCATCAACATCACCCAGTCTGCTCGAGCCGTCGTGTTCATGGGAACACTGACAGCAGGTGGACTTGAGGTTCGAGCTGGTGATGGAGGACTCCAGATCGTCCGAGAGGGCCGAGTCAAGAAGATCGTCCCTGAGGTGTCTCACCTGTCCTTTAACGGTCCCTATGTGGCTTCTCTCGGAATCCCTGTCCTGTACATCACTGAGCGAGCTGTTTTCGAGATGCGAGCTGGAGCTGATGGCGAAGCCCGATTGACTCTGGTGGAGATTGCGCCCGGTGTCGACCTTCAGCGGGACGTTTTGGACCAGTGTAGCACACCCATTGCTGTCGCCCAGGATCTGCGTGAGATGGATGCCCGTCTGTTTCAGGCCGGTCCCCTGCATCTGTAA |
| *Cppct* | ATGCGAAAGGTTCCCATCATCACTGCTGACGAGGCTGCCAAGCTCATCAAGGACGGAGATACCGTTACTACTTCGGGTTTTGTCGGAAACGCTATCCCTGAGGCTCTGGACCGAGCTGTCGAGAAGCGATTCCTCGAGACCGGCGAGCCTAAGAACATTACTTACGTTTACTGTGGATCTCAGGGTAACCGAGACGGACGAGGTGCTGAGCACTTTGCCCATGAGGGCCTGCTCAAGCGATACATTGCTGGACACTGGGCCACCGTTCCCGCTCTGGGAAAGATGGCCATGGAGAACAAGATGGAGGCTTACAACGTGTCCCAGGGAGCCCTGTGCCACCTCTTCCGAGACATCGCCTCGCATAAGCCCGGTGTTTTCACCAAGGTCGGCATCGGAACTTTTATTGACCCTCGAAACGGCGGCGGCAAGGTCAACGACATCACCAAGGAAGACATTGTTGAGCTGGTGGAGATTAAGGGCCAGGAGTACCTCTTCTACCCCGCCTTTCCTATCCACGTGGCTCTGATTCGAGGAACCTACGCCGACGAGTCCGGTAACATCACTTTTGAGAAGGAAGCCGCTCCCCTCGAGGGAACCTCTGTCTGTCAGGCTGTTAAGAACTCCGGCGGAATTGTGGTCGTTCAGGTCGAGCGAGTGGTCAAGGCCGGAACTCTGGACCCCCGACATGTCAAGGTTCCTGGTATCTACGTGGATTACGTTGTGGTCGCTGACCCCGAGGATCACCAGCAGTCGCTGGACTGCGAGTACGATCCCGCCCTCTCTGGCGAGCATCGACGACCTGAGGTTGTGGGAGAGCCCCTGCCTCTCTCGGCTAAGAAGGTCATCGGCCGACGAGGAGCCATTGAGCTGGAGAAGGACGTGGCTGTCAACCTCGGTGTGGGAGCTCCTGAGTACGTGGCTTCTGTTGCTGACGAGGAAGGCATCGTCGATTTCATGACCCTGACTGCCGAGTCTGGAGCTATTGGTGGCGTGCCTGCTGGAGGTGTCCGATTCGGTGCCTCCTACAACGCCGACGCTCTGATTGATCAGGGCTACCAGTTTGACTACTACGATGGCGGAGGTCTGGACCTCTGTTACCTGGGTCTCGCTGAGTGCGATGAGAAGGGCAACATCAACGTGTCCCGATTCGGTCCCCGAATTGCCGGCTGTGGCGGCTTCATCAACATTACCCAGAACACTCCTAAGGTTTTCTTTTGCGGCACCTTCACTGCTGGTGGCCTGAAGGTGAAGATCGAGGACGGCAAGGTCATCATTGTCCAGGAAGGCAAGCAGAAGAAGTTCCTGAAGGCCGTCGAGCAGATCACCTTTAACGGAGACGTTGCCCTCGCTAACAAGCAGCAGGTGACCTACATTACTGAGCGATGTGTCTTCCTGCTCAAGGAAGACGGTCTGCACCTCTCTGAGATTGCTCCTGGCATTGATCTGCAGACCCAGATCCTCGACGTGATGGATTTTGCTCCTATCATTGACCGAGATGCCAACGGCCAGATTAAGCTGATGGATGCTGCTCTGTTTGCTGAGGGTCTGATGGGCCTGAAGGAGATGAAGTCTTAG |
| *Ecpct* | ATGAAACCTGTCAAACCGCCTCGAATCAACGGCCGAGTTCCAGTTCTCTCTGCCCAGGAAGCCGTTAACTACATTCCCGATGAGGCTACCCTCTGTGTCCTTGGCGCTGGAGGAGGCATTCTTGAGGCCACCACGCTGATTACAGCCCTGGCTGACAAGTACAAGCAGACGCAGACTCCCCGAAATCTGTCCATTATCTCTCCCACAGGACTTGGTGATCGAGCTGATCGAGGCATTTCCCCTCTGGCACAAGAGGGACTGGTGAAGTGGGCGCTGTGCGGTCATTGGGGCCAGTCTCCACGAATTAGCGATCTGGCCGAACAGAACAAGATTATTGCCTACAACTACCCTCAGGGTGTGCTTACCCAGACCCTCCGAGCCGCAGCTGCCCATCAACCCGGCATTATCTCCGACATCGGCATTGGAACCTTTGTCGATCCCCGACAGCAGGGCGGCAAGCTGAACGAGGTGACCAAAGAGGACCTCATCAAGTTGGTTGAGTTCGACAACAAGGAGTACCTTTACTACAAGGCCATTGCTCCCGATATTGCCTTCATTCGTGCAACCACCTGCGATTCCGAAGGCTACGCCACTTTTGAGGACGAGGTGATGTATCTCGACGCCCTGGTTATTGCGCAAGCTGTCCACAACAACGGTGGAATCGTGATGATGCAGGTCCAGAAGATGGTTAAGAAGGCCACGCTTCACCCCAAGTCCGTGCGTATCCCCGGTTACCTCGTCGACATCGTGGTCGTTGACCCGGATCAGTCTCAGTTGTATGGTGGCGCCCCAGTCAACCGATTCATCTCTGGCGACTTCACCCTCGACGACTCCACCAAGCTGTCGCTTCCCCTCAATCAGCGGAAGCTTGTCGCTAGACGAGCACTGTTTGAGATGCGGAAAGGAGCGGTCGGAAACGTGGGTGTCGGCATTGCCGATGGTATCGGACTCGTTGCCCGAGAAGAAGGTTGTGCTGACGACTTCATTTTGACCGTCGAGACTGGCCCTATCGGCGGAATCACTTCGCAAGGAATCGCCTTTGGCGCCAATGTCAACACCCGAGCCATCCTTGACATGACGTCCCAGTTTGACTTCTACCACGGAGGAGGTCTGGACGTGTGCTACCTGTCGTTTGCAGAAGTCGACCAGCATGGCAACGTTGGTGTCCACAAGTTCAACGGCAAGATCATGGGAACCGGAGGCTTCATCGACATCTCCGCTACTTCCAAGAAGATCATCTTCTGTGGCACACTCACCGCTGGTTCTCTCAAGACTGAGATTGCTGACGGTAAGCTGAACATTGTGCAGGAGGGCCGAGTCAAGAAGTTCATCCGAGAACTGCCTGAGATCACCTTCAGCGGCAAGATCGCCCTGGAGAGAGGTCTGGATGTGCGGTACATCACAGAGAGAGCTGTGTTTACTCTGAAAGAGGATGGTCTGCACTTGATCGAGATTGCTCCTGGTGTTGACCTGCAGAAGGACATCCTCGACAAGATGGATTTCACTCCCGTGATCTCCCCTGAGCTGAAGCTGATGGACGAGCGACTCTTCATTGACGCTGCCATGGGTTTTGTCCTCCCCGAGGCTGCGCACTAA |
| *Enpct* | ATGACCCACCCCCAGCAGGCCGTTCACGCCGCTTCGCTCCAGAACCCCGAGGCTTTTTGGTCCCATCACGCCCAGCAGCTCCATTGGCACAAGAAGCCCTCGCGAGCCATTGGCCGATCTACCAAGACTCTGGCTTCTGGAGCCTCCCACGAGTCCTGGTCGTGGTTCCCTGACGGAGAGATCTCCACCACTTACAACTGTGTGGATCGACATGTCCTGAACGGCAACGGAGACAACGTGGCCATCATTTGGGATTCTGCTGTCACCGGCAAGAAGGAGAAGTACACTTACCGACAGCTGCTCGACGAGGTCGAGGTTCTGGCTGGTGTCCTCCGAGAGGAGGGCGTTAAGAAGGGAGACGTGGTCATCATCTACATGCCCATGATCCCTGCCGCTCTGATTGGAGCTCTCGCTGTCGCTCGACTGGGTGCTATTCACGCCGCTGTTTTCGGCGGATTTGCCGCTAAGTCCCTGGCTCAGCGAATTGAGGCTGCTCGACCCCGAGCTATCCTCACCGCTTCTTGCGGTATTGAGGGCGCCAAGGGACCCATCGCTTACCGACCTCTGGTGGAGGGCGCTATTGAGGCCTCTTCCTTCAAGCCCGAGAAGGTCCTGATCTGGCAGCGAGACCAGCTCCGATGGAACAACCCTGATAAGCTGGGTGGCCAGCGAAACTGGAACCGACTCGTGAAGTCCGCCCGAATGCGAGGCATTCGAGCTGAGCCCGTGCCTGTCCGATCTACCGACGGACTGTACATCATCTACACTTCCGGTACCACTGGCCTCCCCAAGGGAGTTGTGCGAGAGGCCGGAGGTCACGCTGTGGGTCTGTCTCTCTCCATCAAGTACCTGTTCGACATTCATGGTCCCGGCGATACCATGTTTTGTGCCTCCGACATTGGTTGGGTCGTTGGCCACTCGTACATCCTGTACGCCCCTCTGCTCGTCGGAGCTACCACTGTTCTCTTCGAGGGAAAGCCTGTGGGTACCCCTGACGCTGGTACTTTTTGGCGAGTGGTCGCCGAGCATAAGGCTAACGTCCTGTTCACCGCTCCCACTGCCCTCCGAGCTATTCGAAAGGAGGACCCTGATAACAAGCACTTTGAGAAGGTGGCCGGTGACAACAACCTGCGACATCTCCGAGCCCTGTTCCTCGCTGGCGAGCGATCGGAGCCCTCTATCGTCCGAGCCTACCAGGACCTGCTCACCAAGCACGCCGCTCGAGGAGCTCTGGTTGTGGATAACTGGTGGTCGTCTGAGTCGGGCTCTCCTATTTCCGGACTGGCTCTCCGATCGGCTGTCGGTCGAGTTCCTCCTCGATCGGACGAGTACGATGTGGCCCCCCTGGCTATCCGACCTGGATCTGCCGGTCTCCCCATGCCTGGTTTCGACGTCCGAGTCGTTGACGATGAGGGCAACGAGGTTGCCCAGGGCACCATGGGAAACATTGTGATGGCTACTCCCCTGGCCCCTACCGCTTTCACTCGACTCTTTAACGACGATGAGCGATTCTACAAGGGATACCTGAAGCGATTTGGCGGACGATGGCTCGACACCGGCGACGCTGGTATGATCGACCAGGATGGCTACATTCACGTGATGTCCCGATCGGACGATATCATTAACGTCGCCGCTCACCGATTCTCTACTGGACAGGGTTCCATCGAGCAGGCCATTCTGTCGCACCCCGCCATTGGAGAGGCTTCTGTGGTCGGCATCCCCGACGCCCTGAAGGGACATCTCCCTTTCGCTTTTATCACCCTGAAGCAGTCCGGTGGTAACTCGCCTGCTCGACCTTCTGCTGAGCTGTTCAACTCCGTTAACCGACTCGTGCGAGAGCAGATCGGAGCTATTGCCTCCCTGGGAGGAATGATCCAGGGCCAGGGAATGATTCCCAAGACCCGATCTGGCAAGACTCTCCGACGAGTGCTGCGAGAGCTCGTCGAGAACGGAGCCCGAGGTGAGTTCGAGAAGGAGGTTGCTGTGCCTCCTACCGTGGAGGACCGAGGCGTTGTGGAGGTTGCCCGAGAGAAGGTGCGAGAGTACTTCGAGTCTCAGTCCGGATCGCCCAAGGCTAAGCTGTAG |
| *EcprpE* | ATGTCTTTCTCCGAGTTCTACCAGCGATCTATCAACGAGCCTGAGCAGTTCTGGGCTGAGCAGGCTCGACGAATTGACTGGCAGACCCCCTTCACCCAGACCCTGGACCACTCCAACCCTCCCTTCGCCCGATGGTTCTGTGAGGGCCGAACCAACCTGTGCCACAACGCTATCGACCGATGGCTGGAGAAGCAGCCTGAGGCTCTGGCTCTGATTGCCGTCTCTTCCGAGACTGAGGAAGAGCGAACCTTCACCTTCCGACAGCTGCACGACGAGGTGAACGCCGTCGCTTCTATGCTGCGATCCCTGGGAGTGCAGCGAGGTGACCGAGTGCTGGTCTACATGCCCATGATCGCCGAGGCTCACATTACCCTGCTGGCCTGTGCTCGAATCGGTGCCATTCACTCTGTGGTCTTCGGCGGATTCGCTTCTCACTCCGTGGCCGCTCGAATCGACGACGCCAAGCCCGTGCTGATTGTGTCCGCTGACGCTGGAGCTCGAGGTGGCAAGATCATTCCCTACAAGAAGCTGCTGGACGACGCTATCTCTCAGGCTCAGCACCAGCCCCGACACGTGCTGCTGGTGGACCGAGGCCTGGCTAAGATGGCTCGAGTGTCTGGACGAGACGTCGACTTCGCTTCTCTGCGACACCAGCACATTGGTGCTCGAGTGCCTGTGGCTTGGCTGGAGTCTAACGAGACTTCCTGCATTCTGTACACCTCTGGTACCACCGGCAAGCCCAAGGGAGTGCAGCGAGACGTCGGAGGTTACGCTGTCGCCCTGGCTACCTCCATGGACACCATTTTCGGCGGAAAGGCCGGATCTGTGTTCTTCTGTGCTTCCGACATCGGCTGGGTGGTCGGACACTCCTACATTGTCTACGCTCCCCTGCTGGCCGGCATGGCTACCATCGTGTACGAGGGACTGCCCACCTGGCCTGACTGTGGTGTGTGGTGGACCATTGTCGAGAAGTACCAGGTGTCTCGAATGTTCTCCGCCCCCACCGCTATCCGAGTGCTGAAGAAGTTCCCCACCGCCGAGATTCGAAAGCACGACCTGTCTTCCCTGGAGGTCCTGTACCTGGCTGGAGAGCCTCTGGACGAGCCTACCGCTTCTTGGGTGTCCAACACCCTGGACGTGCCCGTCATCGACAACTACTGGCAGACCGAGTCTGGTTGGCCCATCATGGCCATTGCTCGAGGCCTGGACGACCGACCTACCCGACTGGGCTCTCCCGGTGTCCCCATGTACGGATACAACGTCCAGCTGCTGAACGAGGTGACCGGAGAGCCCTGTGGCGTGAACGAGAAGGGTATGCTGGTGGTGGAGGGTCCTCTGCCTCCCGGTTGCATCCAGACCATTTGGGGCGACGACGGACGATTCGTCAAGACCTACTGGTCCCTGTTCTCTCGACCCGTGTACGCCACCTTCGACTGGGGAATCCGAGACGCTGACGGTTACCACTTCATCCTGGGCCGAACCGACGACGTGATTAACGTCGCCGGACACCGACTGGGTACCCGAGAGATCGAGGAGTCTATTTCTTCTCACCCTGGAGTGGCTGAGGTGGCTGTGGTCGGAGTCAAGGACGCTCTGAAGGGTCAGGTGGCCGTCGCTTTCGTGATTCCCAAGGAGTCTGACTCCCTGGAGGACCGAGACGTCGCCCACTCTCAGGAGAAGGCCATCATGGCTCTGGTGGACTCCCAGATTGGTAACTTCGGCCGACCCGCTCACGTGTGGTTCGTCTCTCAGCTGCCCAAGACCCGATCCGGAAAGATGCTGCGACGAACCATCCAGGCCATTTGTGAGGGTCGAGATCCCGGCGACCTGACCACCATCGACGACCCTGCTTCCCTGGACCAGATTCGACAGGCTATGGAGGAGTAG |
| *SeprpE* | ATGTCTTTCTCCGAGTTCTACCAGCGATCTATCAACGAGCCTGAGGCTTTCTGGGCTGAGCAGGCTCGACGAATTGACTGGCGACAGCCCTTCACCCAGACCCTGGACCACTCTCGACCTCCCTTCGCTCGATGGTTCTGTGGCGGAACCACCAACCTGTGCCACAACGCCGTCGACCGATGGCGAGACAAGCAGCCTGAGGCTCTGGCTCTGATTGCCGTGTCTTCCGAGACTGACGAGGAGCGAACCTTCACCTTCTCTCAGCTGCACGACGAGGTGAACATTGTCGCCGCTATGCTGCTGTCCCTGGGCGTGCAGCGAGGCGACCGAGTGCTGGTCTACATGCCCATGATCGCCGAGGCTCAGATTACCCTGCTGGCCTGTGCTCGAATCGGCGCTATTCACTCTGTGGTCTTCGGTGGCTTCGCTTCTCACTCTGTGGCTGCTCGAATCGACGACGCTCGACCCGCCCTGATTGTGTCTGCTGACGCTGGAGCTCGAGGCGGCAAGATCCTGCCCTACAAGAAGCTGCTGGACGACGCTATTGCCCAGGCTCAGCACCAGCCCAAGCACGTGCTGCTGGTCGACCGAGGTCTGGCTAAGATGGCCTGGGTGGACGGCCGAGATCTGGACTTCGCTACCCTGCGACAGCAGCACCTGGGTGCTTCTGTGCCCGTCGCCTGGCTGGAGTCTAACGAGACTTCCTGCATCCTGTACACCTCCGGCACCACCGGAAAGCCTAAGGGAGTGCAGCGAGACGTCGGCGGATACGCTGTGGCTCTGGCTACCTCTATGGACACCATTTTCGGTGGCAAGGCTGGAGGTGTGTTCTTCTGTGCCTCTGACATCGGTTGGGTGGTCGGCCACTCCTACATTGTCTACGCTCCCCTGCTGGCTGGTATGGCCACCATCGTGTACGAGGGCCTGCCTACCTACCCTGACTGCGGAGTGTGGTGGAAGATTGTCGAGAAGTACCAGGTGAACCGAATGTTCTCCGCTCCCACTGCCATCCGAGTCCTGAAGAAGTTCCCCACCGCCCAGATTCGAAACCACGACCTGTCTTCTCTGGAGGCTCTGTACCTGGCTGGAGAGCCTCTGGACGAGCCTACCGCCTCTTGGGTGACCGAGACTCTGGGCGTGCCCGTCATCGACAACTACTGGCAGACCGAGTCCGGTTGGCCCATTATGGCTCTGGCTCGAGCTCTGGACGACCGACCTTCCCGACTGGGTTCCCCCGGAGTCCCTATGTACGGATACAACGTCCAGCTGCTGAACGAGGTGACCGGAGAGCCCTGTGGTATCAACGAGAAGGGCATGCTGGTCATCGAGGGACCTCTGCCTCCCGGTTGCATCCAGACCATTTGGGGAGACGACGCCCGATTCGTCAAGACCTACTGGTCCCTGTTCAACCGACAGGTGTACGCTACCTTCGACTGGGGCATCCGAGACGCCGAGGGATACTACTTCATTCTGGGTCGAACCGACGACGTGATCAACATTGCCGGCCACCGACTGGGAACCCGAGAGATCGAGGAGTCTATTTCTTCCTACCCTAACGTGGCTGAGGTGGCTGTGGTCGGCATCAAGGACGCTCTGAAGGGACAGGTGGCCGTCGCTTTCGTCATTCCCAAGCAGTCTGACACCCTGGCTGACCGAGAGGCTGCTCGAGACGAGGAGAACGCTATCATGGCCCTGGTGGACAACCAGATTGGACACTTCGGTCGACCCGCTCACGTGTGGTTCGTCTCTCAGCTGCCCAAGACCCGATCCGGCAAGATGCTGCGACGAACCATCCAGGCCATTTGTGAGGGTCGAGATCCCGGCGACCTGACCACCATCGACGACCCTGCTTCCCTGCAGCAGATCCGACAGGCCATTGAGGAGTAG |
| *YlACS2* | ATGTCTGAAGACCACCCAGCCATCCACCCACCCTCCGAGTTCAAGGACAACCACCCCCACTTCGGAGGCCCCCACCTCGACTGTCTGCAGGACTACCACCAGCTGCACAAGGAGTCCATTGAGGACCCCAAGGCCTTCTGGAAGAAGATGGCCAACGAGCTCATCTCCTGGTCAACCCCCTTTGAAACTGTGCGATCTGGCGGCTTCGAGCACGGCGACGTGGCCTGGTTCCCCGAGGGCCAGCTCAACGCCTCCTACAACTGTGTGGATCGACACGCCTTTGCCAACCCCGACAAGCCCGCCATCATTTTTGAGGCCGATGAGCCGGGCCAGGGCCGAATCGTCACCTACGGCGAACTGCTGCGACAGGTGTCTCAGGTCGCAGCCACCCTGCGATCCTTCGGCGTCCAGAAGGGCGATACTGTGGCCGTCTACCTGCCCATGATCCCCGAGGCCATTGTCACTCTGCTGGCCATCACCCGAATTGGCGCTGTCCACTCGGTCATCTTCGCCGGCTTCTCCTCCGGTTCTCTGCGAGACCGAATCAACGACGCCAAGTCCAAGGTTGTCGTCACCACCGACGCCTCCATGCGAGGAGGCAAGACCATCGACACCAAGAAGATTGTCGATGAAGCCTTGCGAGACTGCCCCTCTGTTACCCACACCCTGGTCTTCCGACGAGCAGGTGTCGAGAACCTGGCCTGGACTGAGGGCCGGGACTTCTGGTGGCACGAGGAGGTCGTCAAGCACCGACCCTACCTTGCCCCCGTCCCCGTTGCCTCCGAGGACCCCATCTTCCTGCTTTACACCTCTGGATCCACCGGCACCCCCAAGGGTCTGGCCCACGCTACCGGTGGCTACCTGCTTGGTGCTGCCCTGACCGCCAAGTACGTGTTTGACATCCACGGAGACGACAAGCTGTTCACCGCTGGAGACGTTGGCTGGATCACCGGCCACACCTACGTGCTCTACGGTCCTCTGATGCTCGGAGCCACCACTGTTGTGTTCGAGGGAACCCCTGCCTACCCCTCCTTCTCGCGATACTGGGACATTGTCGACGACCACAAGATCACCCACTTCTACGTGGCTCCCACCGCCCTGCGTCTCCTGAAGCGGGCCGGCACCCATCACATTAAGCACGACCTGTCGTCTCTGCGAACCCTCGGCTCTGTGGGTGAGCCCATTGCCCCCGACGTGTGGCAGTGGTACAACGACAACATTGGCCGAGGCAAGGCCCACATCTGTGACACCTACTGGCAGACCGAGACTGGCTCGCATATCATTGCCCCCATGGCCGGCGTGACCCCCACCAAGCCCGGTTCTGCTTCCCTGCCTGTCTTTGGAATTGATCCCGTTATCATTGATCCCGTGTCTGGCGAGGAGCTCAAGGGTAACAACGTTGAGGGTGTTCTTGCCCTGCGATCTCCCTGGCCCTCCATGGCCCGAACCGTGTGGAACACCCACGAGCGATACATGGAGACCTACCTGCGGCCCTACCCCGGCTACTACTTCACCGGTGATGGTGCTGCCCGAGACAATGACGGCTTTTACTGGATCCGAGGCCGAGTCGACGACGTTGTCAACGTTTCTGGCCACCGTCTTTCCACCGCCGAGATTGAGGCTGCTCTCATTGAGCACGCTCAGGTGTCTGAGTCTGCCGTTGTTGGTGTCCATGACGATCTGACTGGCCAGGCCGTCAACGCCTTTGTGGCTCTCAAGAACCCCGTCGAGGATGTGGACGCTCTGCGAAAGGAGCTTGTTGTGCAGGTGCGAAAGACCATTGGACCCTTTGCTGCTCCCAAGAATGTCATCATCGTGGACGATCTGCCCAAGACTCGGTCTGGCAAGATCATGCGACGAATTCTGCGAAAGGTGCTTGCTGGCGAGGAGGACCAGCTCGGAGACATTTCCACTCTTGCTAACCCCGACGTTGTCCAGACCATCATTGAGGTTGTTCACTCGTTGAAAAAGTAA |
| *RebktB* | ATGACCCGAGAGGTGGTGGTGGTCAGCGGTGTTCGAACCGCCATCGGCACCTTCGGCGGCTCCCTGAAGGACGTCGCCCCCGCTGAGCTGGGCGCTCTGGTTGTCCGAGAGGCCCTGGCCCGAGCTCAGGTGTCTGGTGACGACGTGGGCCACGTCGTCTTCGGCAACGTCATTCAGACCGAGCCCCGAGACATGTACCTGGGACGAGTCGCTGCCGTCAACGGCGGCGTCACCATTAACGCCCCCGCCCTCACCGTGAACCGACTGTGCGGTTCCGGCCTGCAGGCCATCGTCTCCGCCGCTCAGACCATCCTGCTCGGCGACACCGACGTTGCCATCGGCGGCGGTGCCGAGTCCATGTCCCGAGCTCCCTACCTGGCCCCCGCCGCTCGATGGGGTGCTCGAATGGGTGACGCCGGTCTGGTTGATATGATGCTGGGAGCCCTGCACGACCCTTTCCACCGAATTCACATGGGCGTTACCGCTGAGAACGTCGCCAAGGAGTACGACATCTCCCGAGCCCAGCAGGACGAGGCTGCCCTGGAGTCCCACCGACGAGCCTCCGCTGCCATCAAGGCCGGCTACTTCAAGGACCAGATCGTCCCCGTCGTTTCCAAGGGCCGAAAGGGCGACGTCACCTTCGACACCGACGAGCACGTCCGACACGACGCCACCATCGACGACATGACCAAGCTGCGACCCGTCTTCGTCAAGGAGAACGGCACCGTGACCGCCGGAAACGCCTCCGGACTGAACGACGCCGCCGCCGCTGTCGTGATGATGGAGCGAGCCGAGGCCGAGCGACGAGGACTGAAGCCCCTGGCCCGACTGGTCAGCTATGGTCACGCCGGTGTGGACCCCAAGGCTATGGGAATTGGTCCCGTCCCCGCCACCAAGATCGCCCTGGAGCGAGCCGGACTGCAGGTGTCCGACCTGGACGTTATCGAGGCCAACGAGGCTTTCGCTGCTCAGGCCTGCGCCGTTACCAAGGCCCTGGGCCTGGACCCCGCCAAGGTCAACCCCAACGGATCTGGCATCTCCCTGGGCCACCCCATCGGCGCTACCGGTGCTCTGATCACCGTCAAGGCCCTGCACGAGCTCAACCGAGTCCAGGGTCGATACGCCCTGGTCACCATGTGCATCGGAGGCGGCCAGGGCATCGCTGCCATCTTCGAGCGAATCTAA |

**Supplementary Table 3.** Production of OCFAs in obese-L strain with or without acetate. The strains were cultivated in YNBD2P0.5 and YNBD2P0.5A1 medium for 120 hours. Averages and standard deviations were obtained from two replicate experiments.

| Media | Lipid content % (g/g DCW) | | OCFA/total lipids (%) |
| --- | --- | --- | --- |
|  | Total lipids | OCFAs |  |
| YNBD2P0.5 | 18.81 ± 1.03 | 5.48 ± 1.05 | 28.94 ± 4.01 |
| YNBD2P0.5A1 | 17.27 ± 0.62 | 0.65 ± 0.01 | 3.79 ± 0.09 |

**Supplementary Table 4.** Production of OCFAs in yeasts.

|  | Substrate  (g/L) | Biomass (g/L) | Lipid  (g/L) | Lipid content % (w/w) | OCFAs /Total lipids (%) | OCFAs  (g/L) | Reference |
| --- | --- | --- | --- | --- | --- | --- | --- |
| *Trichosporon cutaneum* | Propionate 4 g/L | 1.60 | 0.38 | 23.90 | ~35 | 0.13 | Kolouchova et al. 2015 |
| *Trichosporon cutaneum* | Glucose 20 g/L  Propionate 4 g/L | 1.76 | 0.64 | 36.10 | < 20 | < 0.13 | Kolouchova et al. 2015 |
| *Cryptococcus curvatus* | Propionate 16.5 g/L | 6.7 | 2.28 | 34.1 | 38.7 | 0.88 | Zheng et al.  2012 |
| *Cryptococcus curvatus* | VFA 30 g/L  (A:P:B = 5:15:10*) | 8.24 | 3.83 | 46.47 | 42.3 | 1.37 | Liu et al.  2017 |
| *Yarrowia lipolytica* | Propionate 4 g/L | 3.53 | 0.31 | 8.90 | ~30 | <0.093 | Koluchova et al. 2015 |
| *Yarrowia lipolytica* | Glucose 20 g/L  Propionate 4 g/L | 3.83 | 0.39 | 10.20 | <15 | <0.040 | Koluchova et al. 2015 |
| *Yarrowia lipolytica* | Pentadecane 3 g/L Rhamnolipids | 2.75 | 0.47 | 17.20 | 44.5 | 0.209 | Matatkova et al. 2017 |
| *Yarrowia lipolytica* (Obese) | Glucose 14 g/L  Propionate 4 g/L | 5.53 | 1.36 | 24.76 | 41.9 | 0.57 | Park et al. 2018 |
| *Yarrowia lipolytica* (Obese-LPB) | Glucose 20 g/L  Propionate 5 g/L  Acetate 10 g/L | 7.32 | 2.04 | 27.84 | 64.54 | 1.32 | This study |
| *Yarrowia lipolytica* (Obese-LPB) | Glucose 40 g/L  Propionate 10 g/L  Acetate 20 g/L | 10.52 | 3.01 | 28.56 | 62.07 | 1.87 | This study |

*A, acetate; P, propionate; B, Butyrate.

**Supplementary Figure 1.** Comparison of growth in wild-type and strains overexpressing propionate activating genes in (a) YNBD0.5, (b) YNBP0.5, (c) YNBD0.5P1, and (d) YNBP1A0.5. Averages were obtained from two replicate experiments.

**
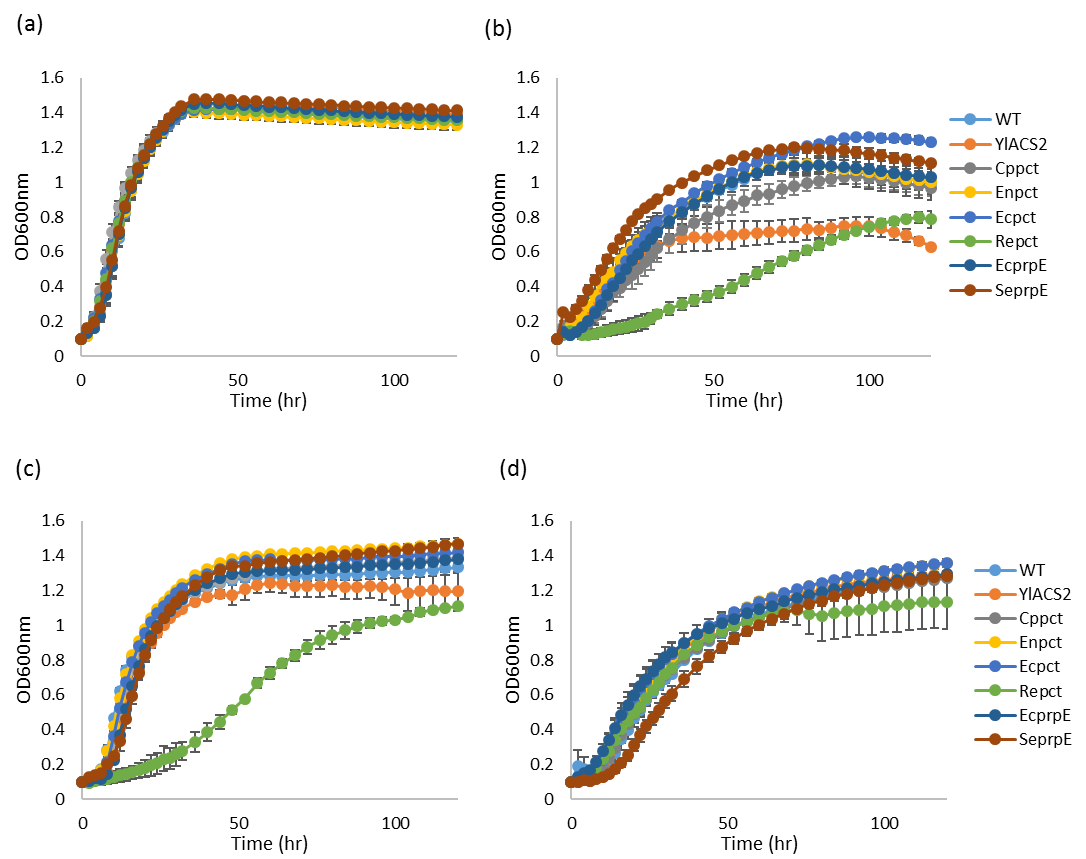
**

**Supplementary Figure 2.** Comparison of growth on different substrates in each strain overexpressing propionate activating genes. •, YNBD0.5; •, YNBP0.5; •, YNBD0.5P1; •, YNBP1A0.5. Averages were obtained from two replicate experiments.


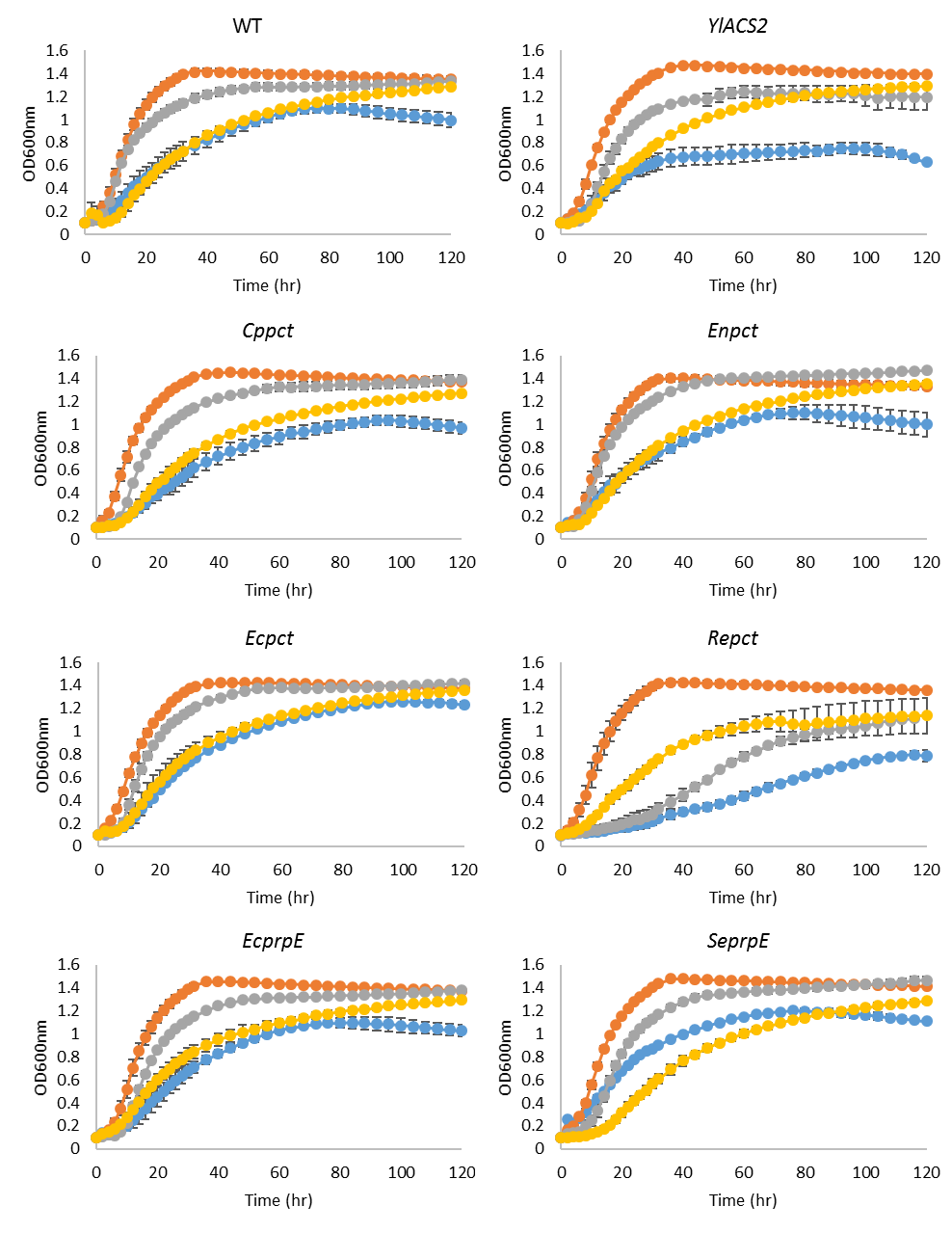


**Supplementary Figure 3.** (a) Lipid profiles (% in total lipids) of obese-L and obese-LP strains, (b) GC chromatogram of obese-L strain (JMY7228, control) and obese-LP (*Repct*) strain (JMY7780). The strains were cultivated in YNBD2P0.5A1 medium for 120 hours. Averages and standard deviations were obtained from two replicate experiments.


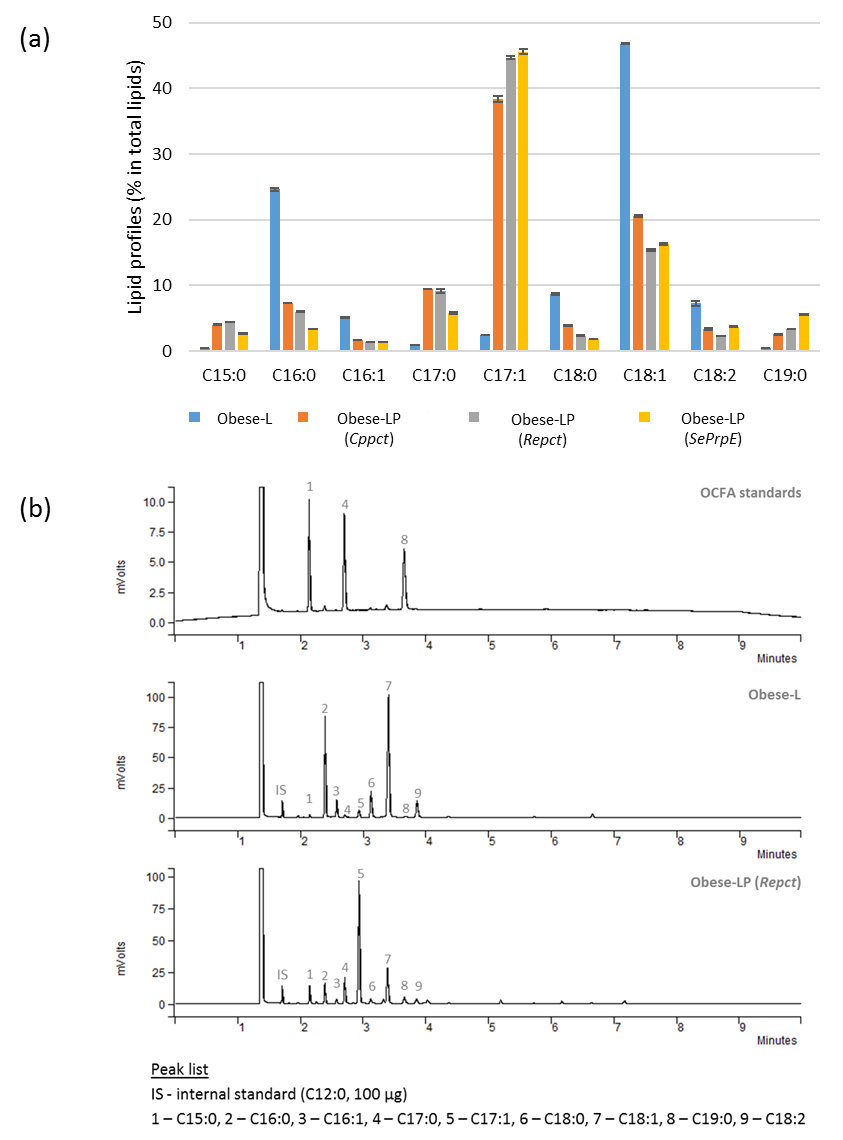


**Supplementary Figure 4.** Substrate consumption of obese strains; (a) control, obese-L (b) obese-LP (*Cppct*), (c) obese-LP (*Repct*), and (d) obese-LP (*SeprpE*). Averages and standard deviations were obtained from two replicate experiments.


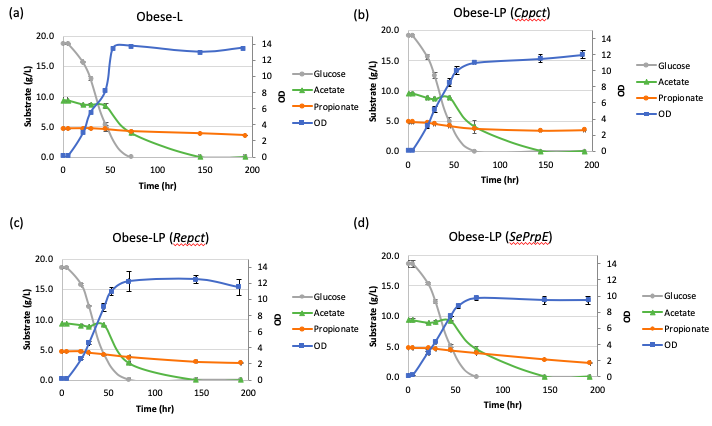

Supplement: Multimedia component 1 [file mmc1.docx]
